# Regulation of α-Ketoglutarate levels by *Myc* affects metabolism and demethylation in porcine early embryos

**DOI:** 10.3389/fcell.2024.1507102

**Published:** 2024-11-26

**Authors:** Ran Ding, Yongfeng Zhou, Qi Zhang, Xiangjie Kong, Qi Li, Sheng Zhang, Yibing Chen, Xinglan An, Ziyi Li

**Affiliations:** ^1^ Key Laboratory of Organ Regeneration and Transplantation of Ministry of Education, First Hospital, Jilin University, Changchun, China; ^2^ Jilin Hospital, Obstetrics and Gynecology Hospital Affiliated to Zhejiang University School of Medicine (Changchun Obstetrics and Gynecology Hospital, Changchun Maternal and Child Health Hospital, Changchun Third Hospital), Changchun, China

**Keywords:** *Myc*, α-KG, metabolism, demethylation, early embryos, pigs

## Abstract

The *Myc* family is essential for cell proliferation, differentiation, and metabolism, particularly in embryonic development and stem cell functions. However, the specific role of *Myc* in porcine early embryonic development is not fully understood. This study observed high *Myc* expression during the four-cell stage of porcine embryos. Inhibition of *Myc* using 10058-F4 impaired embryonic development, disrupted energy metabolism, and increased DNA methylation. Mechanistically, these effects were dependent on α-KG, a TCA cycle intermediate and cofactor for TET demethylation enzymes. Sequencing analysis of four-cell embryos post-*Myc* inhibition revealed downregulation of key metabolic enzymes related to α-KG, such as CS, IDH2, leading to reduced α-KG levels. Supplementation with α-Ketoglutarate (α-KG) mitigated the negative effects of *Myc* inhibition, including lower blastocyst rates, decreased ATP levels, and increased 5 mC levels. In conclusion, *Myc* regulates the expression of key metabolic enzymes during the four-cell stage, influencing early embryonic metabolism and epigenetic reprogramming.

## Graphical Abstract

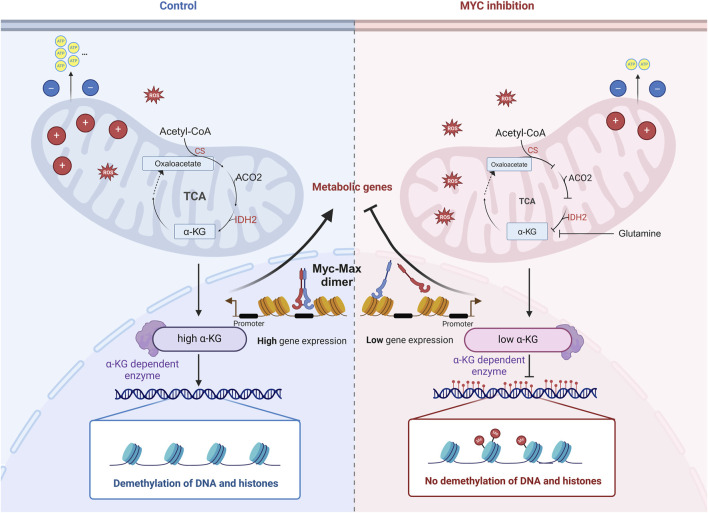

## 1 Introduction

The *Myc* (Formerly known as *c-myc*) is a key member of the *Myc* oncogene family and functions as a universal transcription amplifier. Its extensive gene regulatory functions enable *Myc* to participate in nearly all physiological processes within cells, including cell proliferation, differentiation, apoptosis, and metabolism (Dejure and Eilers, 2017; Knoepfler, 2007; Lin et al., 2012; Rahl et al., 2010). Since the discovery of the *Myc* oncogene in avian viruses in 1982 (Colby et al., 1983), numerous studies have shown that *Myc* oncogene family is highly expressed in approximately 15% of human cancers. This high expression is not due to sequence mutations but rather pathological overexpression (Dang, 2012). Abnormally high *Myc* expression can reprogram cellular metabolism by promoting glycolysis and glutaminolysis (Osthus et al., 2000; Kim et al., 2004; Hsieh et al., 2015; Le et al., 2010), indirectly increasing TCA cycle intermediate levels (Yuneva et al., 2012; Murphy et al., 2013). In cancer cells with high *Myc* expression, there is a severe dependence on mitochondrial oxidative phosphorylation for energy production and macromolecule synthesis (Murphy et al., 2013), indicating *Myc*’s crucial role in metabolic regulation.

*Myc* plays a crucial role in maintaining the self-renewal and differentiation potential of embryonic stem (ES) cells. In terms of self-renewal, *Myc* activates the Wnt/β-catenin signaling pathway to sustain pluripotency. *Myc* recruits the Polycomb Repressive Complex 2 (PRC2) to suppress the expression of Wnt antagonist genes, thereby stabilizing and enhancing β-catenin activity and reinforcing Wnt signaling. Both *Myc* and *N-myc* are downstream targets of the Wnt signaling pathway, forming a self-amplifying positive feedback loop that ensures ES cells remain in an undifferentiated state even in the absence of exogenous stimuli (Hirai et al., 2011). Conversely, upon receiving differentiation signals, *Myc* expression is downregulated, which releases the inhibition on differentiation-related genes. For instance, during the differentiation of neural stem cells into neurons, *Myc* expression diminishes, while neuron-specific transcription factors such as NeuroD and Ngn2 are upregulated (Varlakhanova et al., 2010). Furthermore, an induced pluripotent stem cell (iPSC) study demonstrates that iPSCs generated using four factors exhibit a higher basal glycolytic rate and capacity compared to those generated with three factors (lacking *Myc*). The production of glycolytic metabolites (e.g., acetate, lactate) and glucose consumption are significantly higher in four-factor-induced iPSCs, whereas their oxidative phosphorylation capacity remains similar to that of three-factor-induced iPSCs (Neri et al., 2012).

Studies of embryonic development have shown that *Myc* is crucial for post-implantation embryonic development. By generating mouse embryonic stem cell (ES) lines with homozygous or heterozygous *Myc* mutations and using somatic cell nuclear transfer (SCNT) to produce cloned embryos, their developmental status can be observed. *Myc* homozygous mutant embryos exhibit embryonic lethality between days 9.5 and 10.5 of gestation (Varlakhanova et al., 2010; Fagnocchi et al., 2016). In contrast, chimeric embryos with homozygous *N-myc* mutations, constructed via microinjection, show lethality at approximately day 11.5 (Davis et al., 1993). In pre-implantation embryos, *Myc* family members display distinct temporal expression patterns. *L-myc* is present as maternal mRNA in oocytes and early mouse embryos, gradually degrading as development proceeds, nearly disappearing by the late two-cell stage; Embryonic transcription begins at the eight-cell stage, but expression levels remain low and unstable (Domashenko et al., 1997) *L-myc* conditional knockout embryos can develop normally to the organismal stage, suggesting a limited role for *L-myc* in early embryonic development. *N-myc* expression is undetectable or low in pre-implantation stages of mouse and pig embryos (Domashenko et al., 1997). In *Xenopus* oocytes, *Myc* protein levels are 10,000 to 100,000 times higher than in somatic cells and begin nuclear translocation in cleavage embryos post-fertilization to support rapid cleavage (Gusse et al., 1989). Using a single-cell sequencing technique not reliant on transcript polyadenylation status, sequencing of human and mouse zygotes revealed zygotic genome activation within 4 h post-fertilization, with these genes potentially regulated by *Myc* (Perry et al., 2023; Asami et al., 2023). *Myc*’s presence as a maternal protein in mature mouse oocytes has been observed, though its precise mechanistic role remains unclear. In mouse embryos, endogenous *Myc* shows significant transcriptional upregulation during the two-cell stage. Knockdown of *Myc* using antisense oligonucleotides (ASO) or small interfering RNA (siRNA) results in two-cell stage arrest (Perry et al., 2023; Paria et al., 1992). A study on failed human IVF embryos found that embryos arrested at the four to eight-cell stages exhibit senescence characteristics, including cell cycle arrest and downregulation of MYC protein and p53 activity (Yang et al., 2022). These findings indicate that *Myc* is essential for pre-implantation embryonic development.

Although there has been substantial research on *Myc* in pre-implantation embryonic development, most studies have focused on observing the developmental outcomes following *Myc* knockout, with relatively few addressing the underlying molecular mechanisms. Our previous transcriptomic analysis of porcine embryos, those cloned via SCNT and derived from *in vivo* fertilization, identified *Myc* as the most significantly differentially expressed transcription factor. At the four-cell stage, *Myc* is underexpressed in SCNT embryos compared to its high expression in *in vivo* fertilized embryos (Zhai et al., 2022). Currently, there is no research on the role of *Myc* in pre-implantation porcine embryonic development. Given *Myc*’s critical role in various physiological functions, we hypothesize that *Myc* also plays a significant role in pre-implantation porcine embryo development. Therefore, this study will use an *in vitro* fertilized embryo model to explore how *Myc* influences early porcine embryo development, aiming to provide new insights and approaches for overcoming the inefficiency of SCNT embryos and constructing animal models for human diseases.

## 2 Results

### 2.1 Spatiotemporal expression and function of *Myc* in early embryos

**FIGURE 1:**
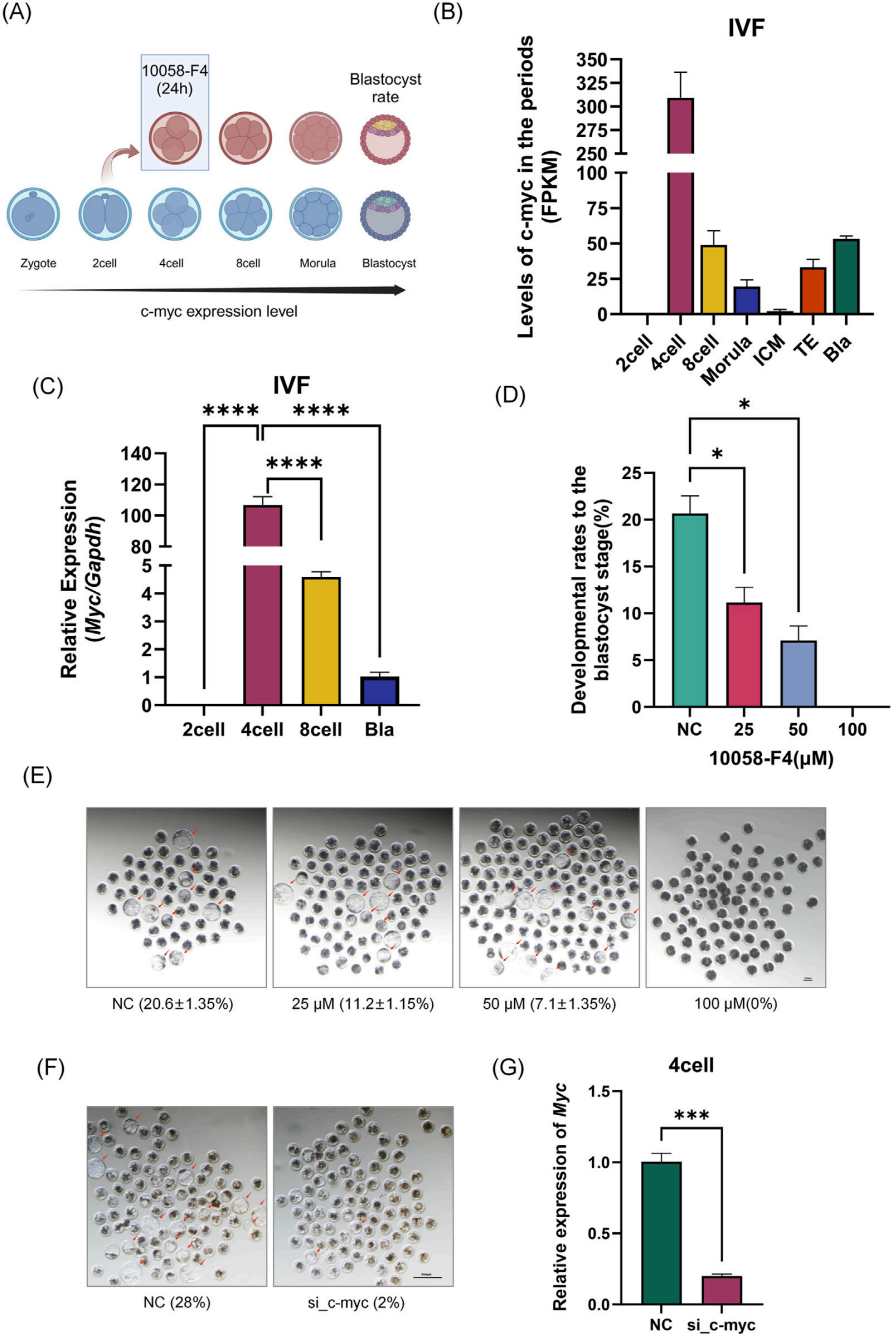
Spatiotemporal Expression and Function of Myc in Early Embryos. **(A)** Experimental Schematic Diagram. **(B)** Expression levels of *Myc* from the two-cell embryo to the blastocyst stage in early porcine embryos. GSEA ID: GSE139512. **(C)** Analysis of *Myc* expression in porcine IVF embryos at the two-cell, four-cell, eight-cell, and blastocyst stages by qPCR. **(D)** Embryo development status after treatment with different concentrations of 10058-F4 (DMSO) and siRNA microinjection (n > 50). **(F)** Changes in blastocyst rate after *Myc* knockdown by small interfering RNA (The siRNA sequences are provided in the supplementary file). **(G)** siRNA interference efficiency detection, with *Myc* expression measured in four-cell embryos after microinjection following IVF. Data represent the mean of three independent experiments, with n representing the sample size for each experiment. Error bars represent standard deviation (SD). *P < 0.05, **P < 0.01. IVF: *In Vitro* Fertilization.

Using the GEO database and quantitative PCR, we validated our lab’s previous sequencing data (Figure 1). As shown in Figures 1B, C, the expression trend of *Myc* aligns with our earlier results, which indicated that *Myc* is highly expressed in four-cell embryos. In four-cell embryos, *N-myc* is only 1.3% of *Myc* (Supplementary Figure 1I). *Myc* mRNA was undetectable in the MII oocytes and two-cell embryos but significantly increased in four-cell embryos (P< 0.01), reaching 23 times the level of GAPDH and five times that of eight-cell embryos (P< 0.01), before rapidly declining thereafter. We further validated MYC protein levels at this stage. As shown in Supplementary Figure 1A, due to the lack of specific antibodies, MYC protein was detected in 1500 MII oocytes and 400 four-cell embryos, providing direct evidence of MYC protein presence in four-cell embryos.

Next, we employed the small molecule inhibitor 10058-F4 and siRNA to interfere with *Myc* (hereafter referred to as MYCi), which resulted in a significant decline in embryonic development capacity (blastocyst rate). As illustrated in Figures 1D–F, increasing doses of 10058-F4 led to a gradual decrease in the blastocyst rate. At a dose of 25 μM, a statistically significant reduction was observed (P = 0.033); at a dose of 50 μM, more than half of the embryos failed to develop, with a blastocyst rate of only 7.1% ± 1.35%, which was significantly different from the control group (20.6% ± 1.35%, P = 0.016). At a dose of 100 μM, all embryos arrested before reaching the morula stage. To further confirm the effect of the inhibitor, we utilized microinjection of siRNA to interfere with *Myc* mRNA. As shown in Figures 1F, G, *Myc* mRNA levels in four-cell embryos decreased by over 82%, indicating substantial interference efficiency. The blastocyst rate also dropped from 28% in the control group (NC) to 2%, consistent with the results obtained from inhibitor treatment.

These findings suggested that the high expression of *Myc* in four-cell embryos was crucial for early embryonic development.

### 2.2 Inhibition of *Myc* leads to abnormal epigenetic modifications

**FIGURE 2:**
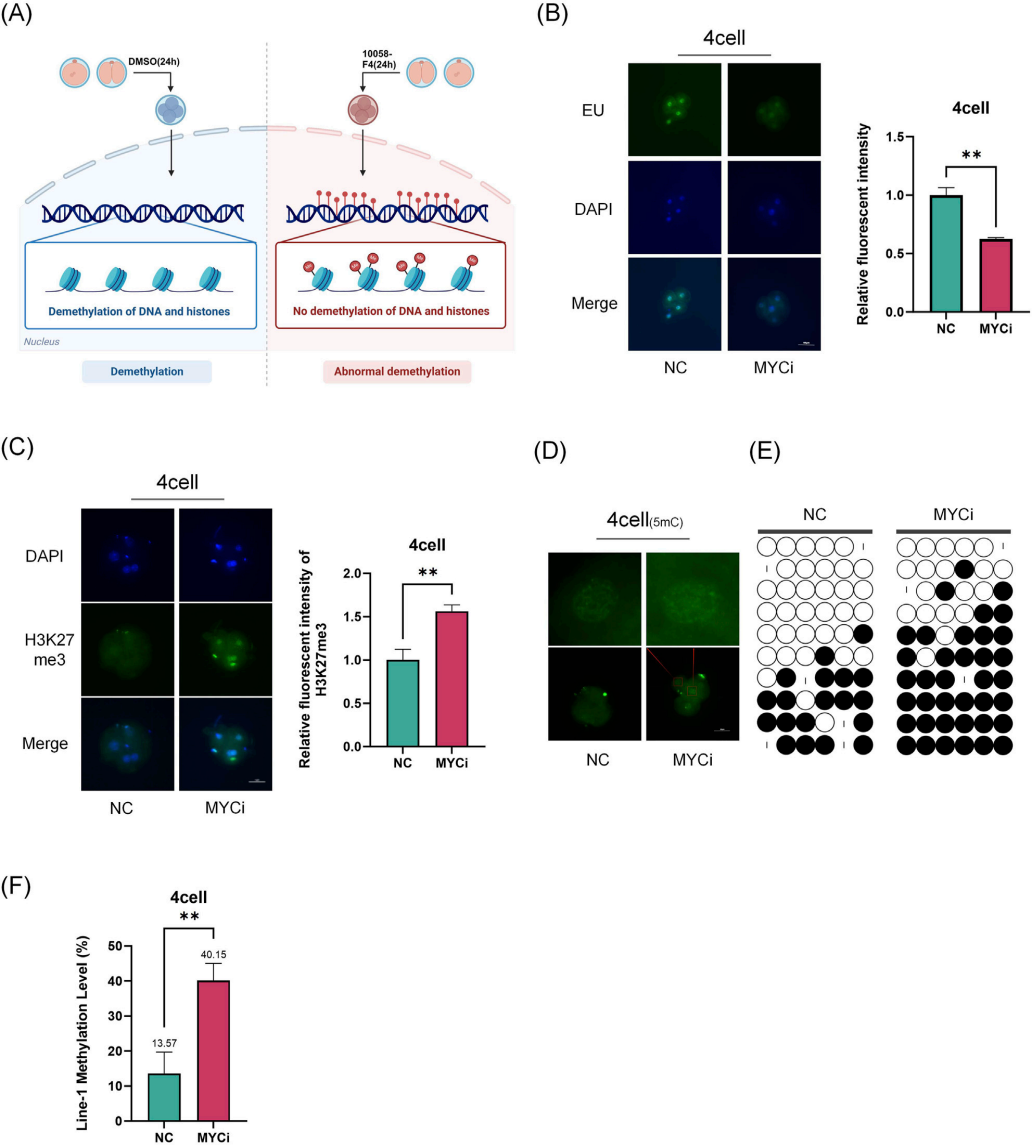
Inhibition of *Myc* Leads to Abnormal Epigenetic Modifications. **(A)** Experimental Schematic Diagram. **(B)** Staining of nascent RNA, representative EU staining images of the MYCi group and control group at the four-cell stage, with P = 0.0045. **(C)** Immunofluorescence of H3K27me3 (green) in four-cell embryos (P = 0.0081), with cell nuclei (blue) stained by DAPI. **(D)** Immunofluorescence of DNA methylation (green) at the four-cell stage. **(E)** Analysis of LINE-1 methylation levels using bisulfite sequencing PCR (BSP) assay. Each base was sequenced more than ten times, and the CpG island methylation rate was statistically analyzed (P = 0.0077). **(F)** LINE-1 methylation rate was used to evaluate the overall genomic methylation levels.

During the four-cell embryo, porcine embryos undergo maternal-to-zygotic transition and epigenetic reprogramming. Our results showed that the high expression of *Myc* at this stage suggesting its essential role in these processes (Figure 2). Using EU staining, we found that newly synthesized RNA decreased by over 50% in the *Myc* inhibition group (MYCi group, Figure 2B), highlighting *Myc*’s vital importance in transcription regulation. Our previous research showed that *Myc* inhibition reduces ZGA-related gene expression (Asami et al., 2023).

Subsequently, we further investigated the changes in epigenetic modifications related to transcriptional activity in the MYCi group. Using immunofluorescence, we observed the levels of H3K27me3 and DNA methylation in four-cell embryos. The results showed a significant increase in H3K27me3 and DNA methylation levels in the MYCi group (Figures 2C, D). LINE-1 is a widely distributed repetitive sequence in the genome. The assessment of LINE-1 methylation levels using bisulfite conversion can serve as a representative measure of the overall genomic methylation status (Xu et al., 2020; Yang et al., 2004). To assess global DNA methylation levels, we examined LINE-1 methylation (Tran et al., 2019) and found that LINE-1 methylation levels were higher in the MYCi group compared to the NC group (40.4% ± 10% VS. 22.8% ± 12.8%) (Figures 2E, F). Additionally, we evaluated the modifications of H3K4me3 and H3K9me3 in four-cell embryos and found no significant differences. Moreover, the difference in H3K27me3 modification disappeared at the eight-cell stage (Supplementary Figure 1B).

These results suggested that *Myc* inhibition regulated gene expression during early embryonic development by affecting DNA and statuses.

### 2.3 Inhibition of *Myc* disrupts gene expression patterns in early embryos

**FIGURE 3:**
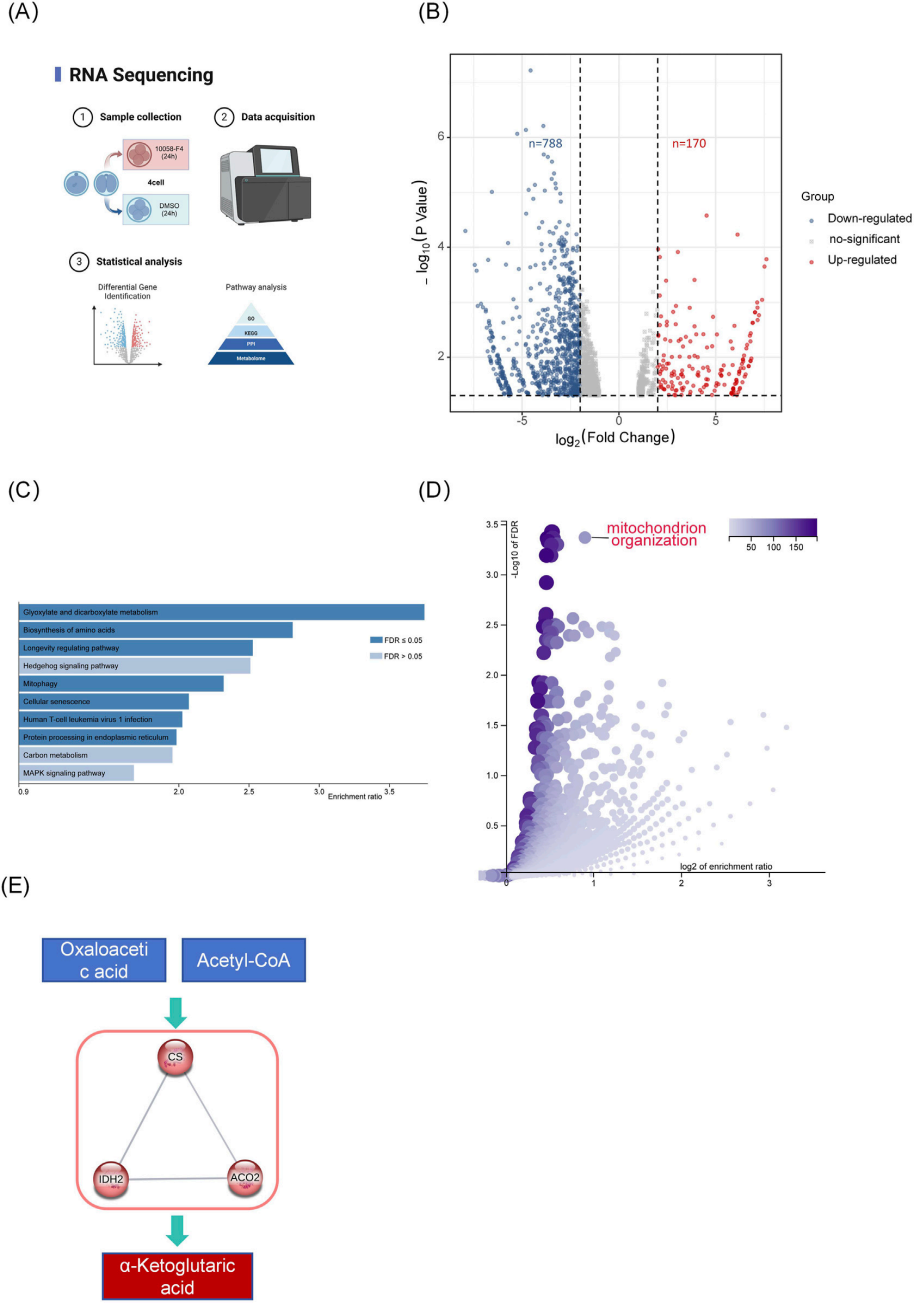
Inhibition of *Myc* Disrupts Gene Expression Patterns in Early Embryos. **(A)** Experimental Schematic Diagram. **(B)** The volcano plot displays the differentially expressed genes (DEGs) between the MYCi group and the control group. Each point represents a gene; red points indicate upregulated genes, while blue points indicate downregulated genes. Genes with p< 0.05 and log2 Fold Change >1 were considered differentially expressed. **(C, D)** KEGG and GO pathway analysis of differentially expressed genes (MYCi vs. control) using WebGestalt, with the *Homo sapiens* database selected for analysis. **(E)** Protein-protein interaction (PPI) network of genes involved in the top-ranked KEGG pathway, “Glyoxylate and dicarboxylate metabolism,” created using STRING-db with “high confidence” and “K-means clustering” parameters.

To further investigate *Myc*’s role in four-cell embryos, we conducted transcriptome sequencing analysis of embryos at this stage (Figure 3). The results showed 788 downregulated genes and 170 upregulated genes in the MYCi group compared to the control group (MYCi vs. control, Figure 3B). This is consistent with the role of *Myc* as a transcription factor that generally promotes gene expression, resulting in the downregulation of most genes upon *Myc* inhibition.

KEGG and GO pathway analysis of differentially expressed genes (DEGs) indicated a close association with energy metabolism. As shown in Figure 3D, GO pathway analysis of differentially expressed genes (DEGs) revealed significant enrichment in mitochondrial organization and several processes related to the positive regulation of transcription and translation, such as positive regulation of transcription, DNA-templated, and positive regulation of RNA biosynthetic process. The enrichment in mitochondrial organization suggests a potential link to cellular energy dynamics.

KEGG analysis revealed that five out of the top ten pathways were related to metabolic processes, including glyoxylate and dicarboxylate metabolism, fructose and mannose metabolism, and biosynthesis of amino acids. Protein interaction network analysis of genes enriched in the KEGG pathway for glyoxylate and dicarboxylate metabolism clustered around key TCA cycle enzymes such as CS, ACO2, and IDH2 (Figure 3E).

### 2.4 *Myc* Inhibition caused mitochondrial dysfunction

**FIGURE 4:**
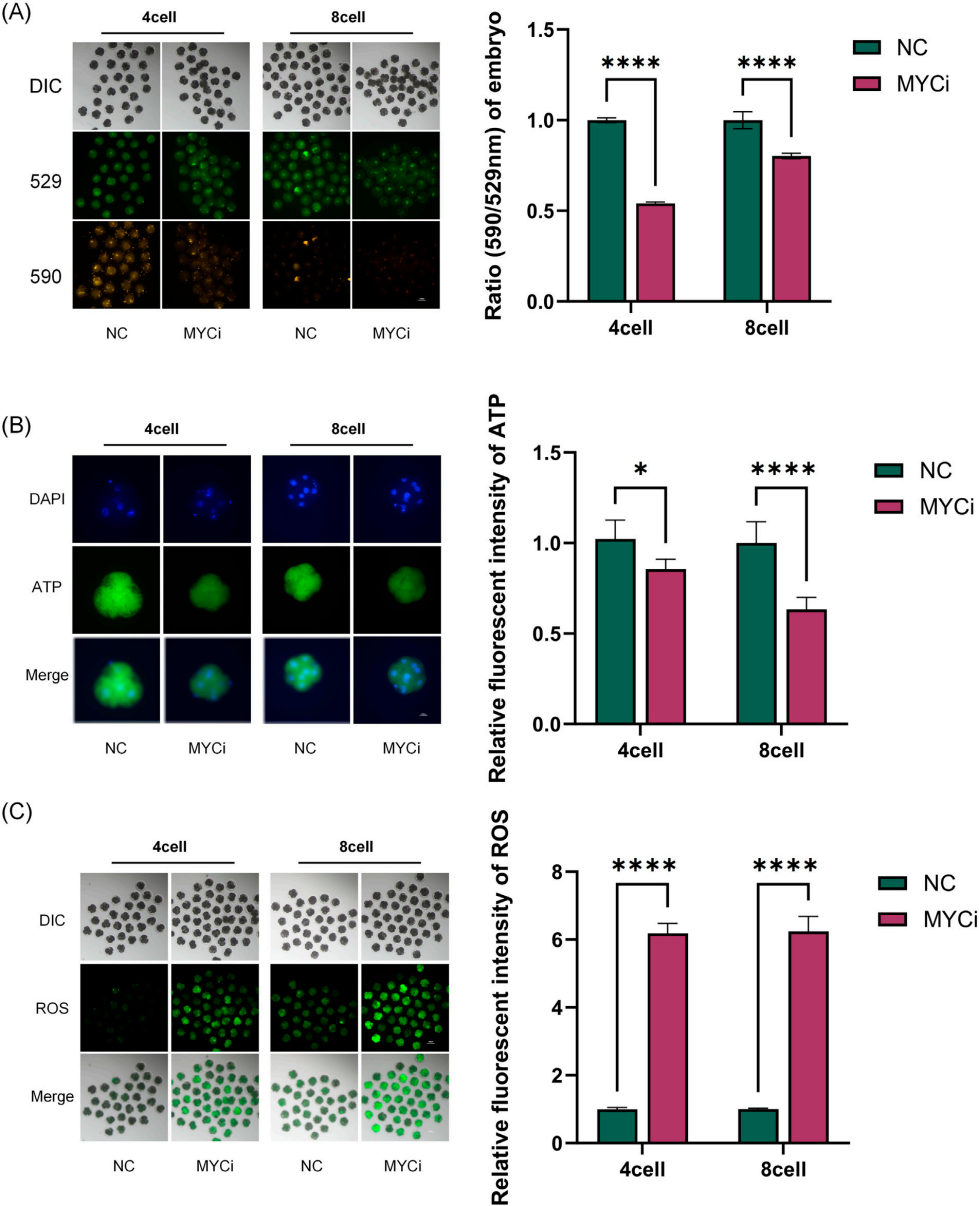
MYCi Group Exhibited Mitochondrial Dysfunction and Enhanced Oxidative Stress **(B)** Embryos were treated with 50 μM 10058-F4 (DMSO) for 24 h, and MMPl was detected in four-cell or eight-cell embryos (P < 0.0001, n > 25). 529 refers to the maximum emission wavelength of JC-1 monomers, while 590 refers to the maximum emission wavelength of aggregated JC-1. The ratio of 590:529 can be used to characterize the mitochondrial membrane potential. **(C)** ATP content detection. The MYCi group exhibited a decrease in ATP content at the four-cell stage (P = 0.0012, n ≥ 8); the decrease in ATP content was more pronounced at the eight-cell stage (P < 0.0001, n > 10). **(D)** ROS detection showed that the MYCi group had significantly higher reactive oxygen species (ROS) levels than the control group at both the four-cell and eight-cell stages (P < 0.0001, n > 20). n represents the sample size for each experiment.

We assessed mitochondrial membrane potential (MMP), ATP, and reactive oxygen species (ROS) levels as macro metabolic indicators (Figure 4). As shown in Figure 4A, the MYCi group exhibited significant reductions in MMP in both four-cell and eight-cell embryos compared to the control group. Embryonic ATP content in the MYCi group was slightly lower in four-cell embryos and halved compared to the control group in eight-cell embryos (Figure 4B). Conversely, ROS levels were significantly higher in the MYCi group in both four-cell and eight-cell embryos (Figure 4C). This indicated that *Myc* inhibition caused abnormalities in mitochondrial energy synthesis and elevated ROS due to the upregulation of genes associated with abnormal oxidative phosphorylation.

### 2.5 Reduced α-KG levels due to *Myc* inhibition contributed to abnormal embryonic development

**FIGURE 5:**
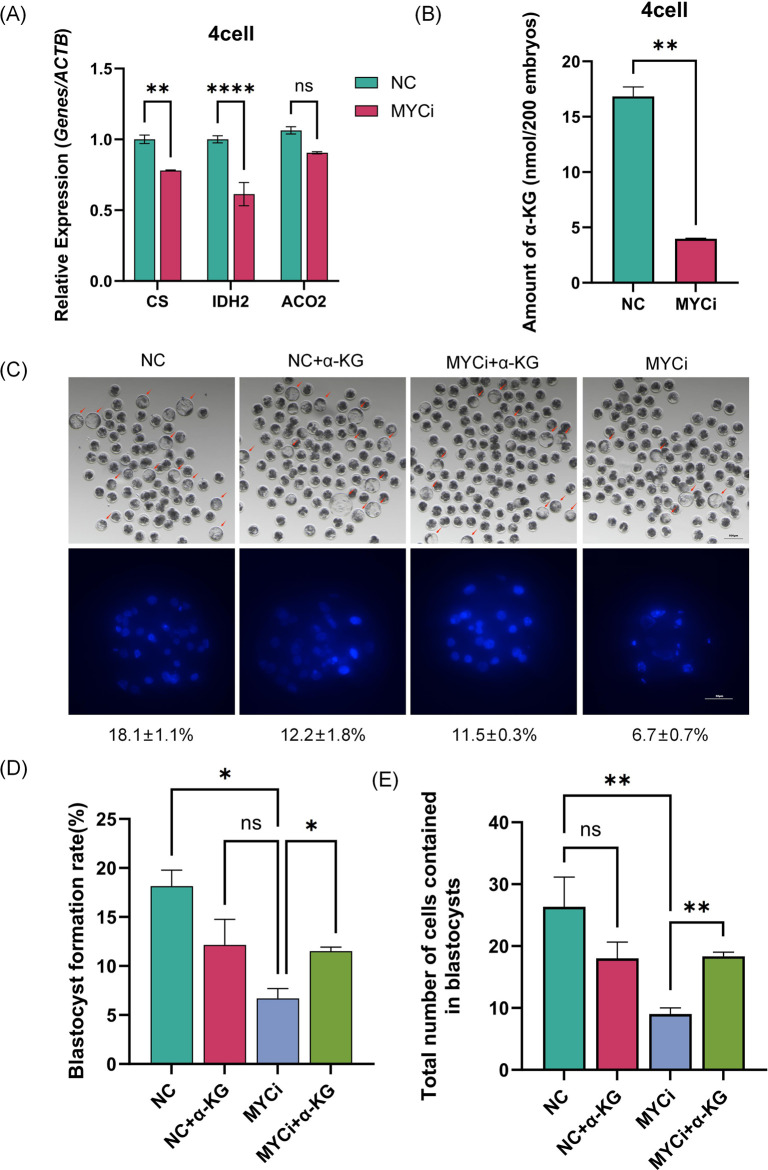
Inhibition of *Myc* Reduced α-KG Levels and Expression of Related Metabolic Enzymes. **(A)** qPCR detection of α-KG-related metabolic enzymes in four-cell stage embryos treated with DMSO or 10058-F4. The expression of CS (p = 0.0048) and IDH2 (p < 0.0001) increased significantly, while the expression of ACO2 decreased but was not statistically significant. **(B)** Quantification of total α-KG in embryos using a colorimetric assay, with at least 300 IVF four-cell embryos per group and an extended reaction time of 90 min (P = 0.0014). **(C)** Assessment of embryo development and total blastocyst cell count after α-KG supplementation (n > 100). Nuclei were stained with DAPI (blue) (n > 7). **(D, E)**: Statistical analysis of blastocyst rate and total blastocyst cell count in the supplementation experiments (n > 5). RT-qPCR normalization was performed using ACTB as the reference gene.

We measured the expression levels of three metabolic enzymes, CS, IDH2, and ACO2, identified in Figure 3E. As shown in Figure 5, all three enzymes were downregulated in the MYCi group, with CS and IDH2 showing significant statistical differences. Considering that CS, ACO2, and IDH2 are responsible for generating α-Ketoglutarate (α-KG) in the TCA cycle, we collected 500 four-cell embryos to measure α-KG content in the MYCi group. As shown in Figure 5B, α-KG content in the MYCi group decreased by more than two-thirds compared to the control group.

Subsequently, we added α-KG to the MYCi group culture system to observe embryonic development. As shown in Supplementary Figures 1D–H, 2 mM α-KG showed the best rescue effect at 25 and 50 μM inhibitor concentrations. Using a 50 μM inhibitor concentration for further testing, the blastocyst rate in the MYCi group dropped from 18.1% ± 1.1% to 6.7% ± 0.7% (Figure 5D, P = 0.0135), indicating effective inhibition. The MYCi+α-KG group showed a significant recovery in the blastocyst rate to 12.2% ± 1.8%, compared to 6.7% ± 0.7% in the MYCi group (P = 0.024), showing a good rescue effect. The NC+α-KG group also showed a slight decrease in the blastocyst rate, likely due to the addition of high concentration 2 mM α-KG.

We then evaluated the total cell number of blastocysts in each group, and the trend was consistent with the aforementioned changes in blastocyst rate. The average total cell number in the MYCi+α-KG group (22) showed a significant increase compared to the MYCi group (12) (Figure 5E).

### 2.6 Supplementation with α-KG effectively alleviated metabolic and epigenetic disruptions induced by *myc* inhibition

**FIGURE 6:**
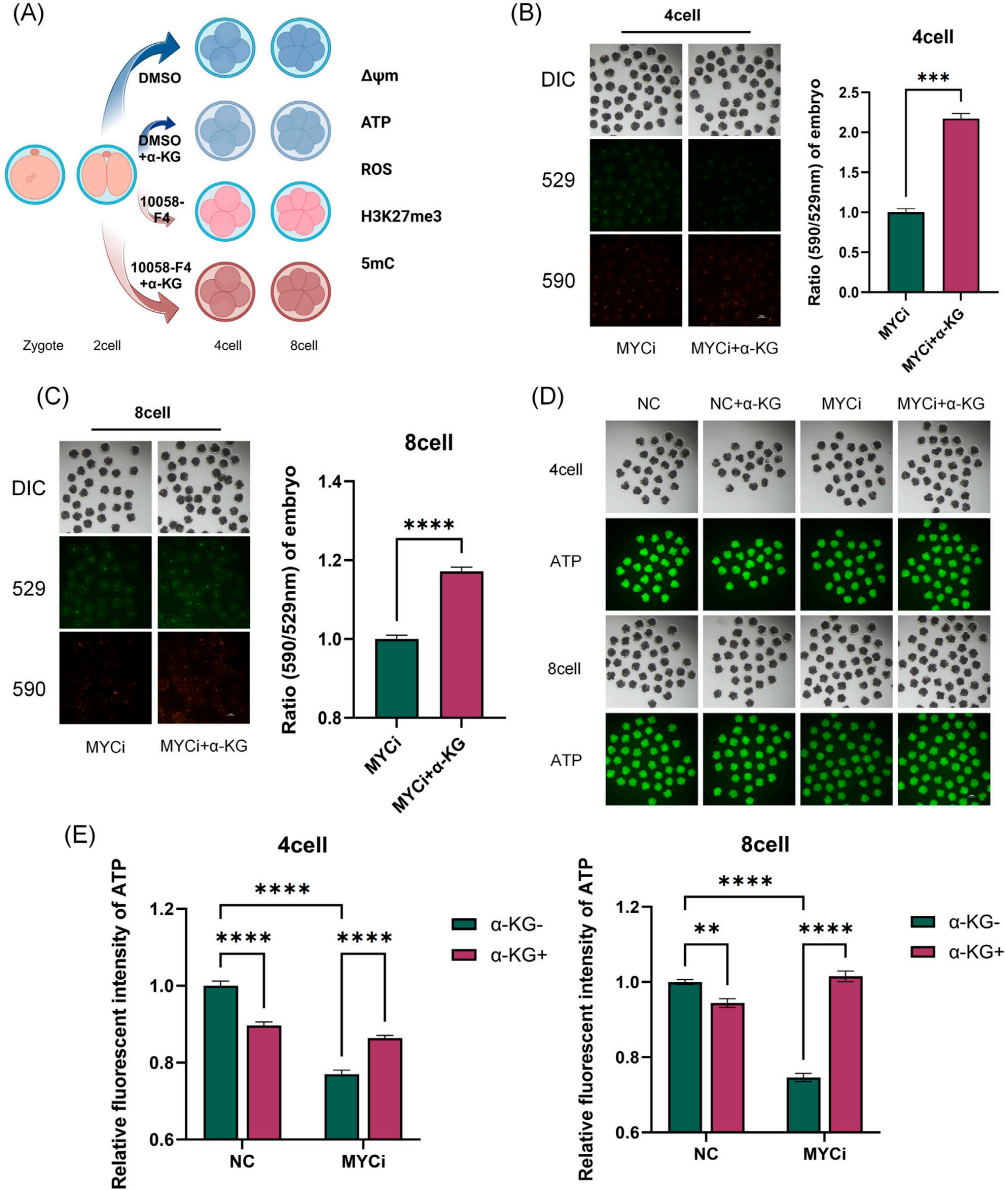
Inhibition of *Myc* with 10058-F4 and α-KG Supplementation Affected Mitochondrial Function, ATP Levels, and ROS Levels. **(A)** Experimental Schematic Diagram. **(B, C)** MMP in four-cell or eight-cell embryos was detected 24 h after treatment with DMSO or 10058-F4, followed by supplementation with 2 mM α-KG (n > 30, p < 0.001). **(D, E)** ATP content detection in four-cell or eight-cell embryos across different groups (n > 20, p < 0.0001).

**FIGURE 7:**
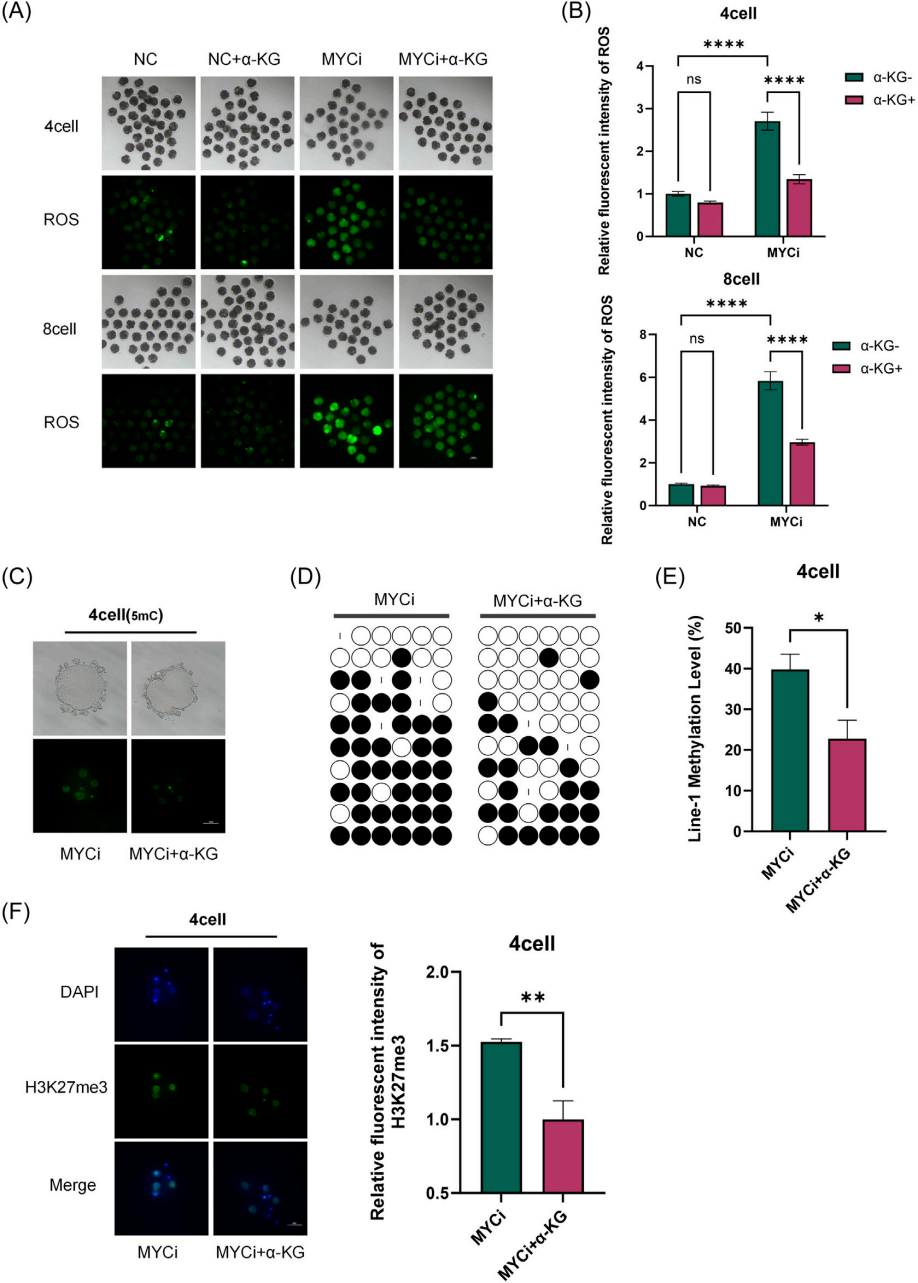
Addition of α-KG Mitigated the Increase in Methylation Modifications Caused by *Myc* Inhibition. **(A, B)**: ROS content detection in four-cell or eight-cell embryos across different groups (n > 30, p < 0.0001). **(C)**: DNA methylation levels (green) in four-cell embryos were assessed after 24 h of treatment with DMSO or 10058-F4, followed by supplementation with 2 mM α-KG. **(D, E)**: Bisulfite sequencing PCR (BSP) analysis of LINE-1 methylation levels. Each base was sequenced more than ten times, and the CpG island methylation rate was statistically analyzed (P = 0.0104). **(F)**: H3K27me3 Immunofluorescence Staining.

The α-KG acts as an intermediate in the TCA cycle, participating in cellular energy metabolism, and as a key cofactor for DNA demethylases, playing an important role in epigenetic modifications. Therefore, in the rescue experiments, we also assessed whether the addition of α-KG improved the abnormal metabolic indicators and DNA methylation levels in the MYCi group (Figures 6, 7).

As shown in Figures 6A–E, 7A, B, compared to the MYCi group, the addition of α-KG significantly improved MMP and ATP content in four-cell and eight-cell embryos, and significantly reduced ROS levels. Specifically, ATP content in the MYCi group was significantly lower than in the normal group, confirming the inhibitor’s effectiveness. In the NC+α-KG group, ATP content decreased, while adding α-KG to the MYCi group significantly increased ATP levels. For ROS detection, ROS levels in the MYCi group were three times higher than those in the normal group, confirming the inhibitor’s effectiveness. Adding α-KG to the NC group did not cause significant changes, but adding α-KG to the MYCi group significantly reduced ROS levels.

In four-cell embryos, we investigated the effects of adding α-KG to the MYCi group in response to the previously observed abnormal elevation of 5 mC and H3K27me3 modifications. Initially, we utilized immunofluorescence techniques to compare the DNA methylation levels between the MYCi group and the MYCi+α-KG group (Figure 7C). The results revealed a notable decrease in DNA methylation levels in the MYCi+α-KG group compared to the MYCi group. To obtain more precise measurements, we further employed bisulfite sequencing to delve deeper into the methylation levels of LINE-1 (Figures 7D, E). The data indicated that the LINE-1 methylation level in the MYCi+α-KG group was significantly reduced from 40.4% ± 10% in the MYCi group to 22.8% ± 12.8%, a change that was statistically significant.

Concurrently, we also observed a marked reduction in H3K27me3 modification levels in the MYCi+α-KG group compared to the MYCi group (Figure 7F). Collectively, these experimental results suggest that the addition of α-KG substantially alleviated the abnormal elevation of DNA and histone methylation levels caused by *Myc* inhibition.

## 3 Discussion

It is well-established that *Myc* plays a critical role in the normal development of pre-implantation embryos, as demonstrated in various species such as mice and *Xenopus* (Paria et al., 1992; Etard et al., 2005; Yang et al., 2022; Zhai et al., 2022; Yamamoto et al., 2023). Our findings extend this understanding by showing that *Myc* is also crucial for the early development of porcine pre-implantation embryos. *Myc* regulates the expression of rate-limiting enzymes such as CS and IDH2, which in turn influences α-KG levels in four-cell stage embryos, thereby modulating embryonic metabolism. Additionally, α-KG, as a cofactor for TET family and JMJC domain-containing demethylases, significantly affects the demethylation processes in early embryos (Tran et al., 2019).

Studies showed that the molecular mechanisms of Myc’s regulation of metabolism in cancer cells were extensively investigated and elucidated (Dejure and Eilers, 2017). *Myc* can regulate the expression of genes related to glycolysis, glutamine metabolism, and fatty acid synthesis, thereby promoting metabolic reprogramming. For instance, the Myc mediated “glutamine addiction mechanism” involves the inhibition of miR-23a and b, leading to the upregulation of GLS1 expression, which promotes glutamine metabolism (Gao et al., 2009). Glutamine undergoes deamination twice to form α-KG, entering the TCA cycle–currently the known direct link between *Myc* and α-KG. Although we did not observe a reduction in GLS1 expression in the MYCi group, our study supports *Myc*’s role in early embryonic metabolic regulation. Specifically, *Myc* inhibition resulted in decreased MMP, reduced ATP levels, increased ROS, and abnormal lipid metabolism in early embryos. RNA-seq analysis revealed that these metabolic abnormalities might be due to reduced expression of enzymes such as CS, IDH2, and ACO2. RT-qPCR confirmed the accuracy of the sequencing results, showing that the reduced expression of these enzymes could lead to lower α-KG levels. Chemical quantification showed a significant reduction in α-KG levels in MYCi group embryos.

Adding α-KG to the *in vitro* culture environment generally supports embryonic development. Treating mouse IVF embryos with 150 μM α-KG and pyruvate improved blastocyst rates and birth rates (Choi et al., 2019); adding 20 μM α-KG to porcine oocyte *in vitro* maturation enhanced oocyte quality (Chen et al., 2022). The role of α-KG in improving embryonic development has various explanations, typically suggesting that α-KG supplementation enhances mitochondrial activity, reduces oxidative stress, and decreases apoptosis. Similarly, in our study, adding α-KG to the MYCi group significantly improved embryonic development and blastocyst quality, alleviating metabolic abnormalities (increased MMP, higher ATP levels, and reduced ROS). However, the optimal concentration of α-KG in our rescue experiments was 2 mM, indicating that α-KG might have additional roles beyond improving metabolism.

It is reported that the L-2-HG to α-KG ratio significantly decreased from approximately sixfold to less than onefold during the transition from zygote to blastocyst in mouse early embryonic metabolomics (Zhao et al., 2021). L-2-HG, a competitive inhibitor of α-KG-dependent dioxygenases, may facilitate the removal of specific histone methylation marks such as H3K4me3 during early embryonic development (Zhao et al., 2021). In our study, we initially identified various histone methylation modifications through immunofluorescence (see Supplementary Figure 1B). The results indicated that only H3K27me3 was significantly increased at the four-cell embryo stage, and this difference dissipated by the eight-cell stage. Subsequently, we evaluated the global genomic methylation levels. We employed bisulfite conversion PCR to assess LINE-1 methylation levels, using it as a representative marker for overall genomic methylation status. We observed a significant increase in 5 mC levels in *Myc*-overexpressing four-cell embryos, which could be alleviated by α-KG supplementation. This suggests that transient upregulation of *Myc* in four-cell embryos may elevate α-KG levels, facilitating demethylation processes and thereby aiding in the reacquisition of totipotency by the embryos. Due to the lack of appropriate methodologies, we were unable to evaluate changes in L-2-HG concentrations during the early embryonic development of pigs. Currently, it is known that L-2-HG is primarily produced by mutant isocitrate dehydrogenase (Sulkowski et al., 2017). The enzyme L-2-hydroxyglutarate dehydrogenase, responsible for the degradation of L-2-HG, is undetectable during early embryonic development. If L-2-HG is present in early porcine embryos, it remains unclear which enzyme catalyzes its production. However, changes in the expression of L-2-hydroxyglutarate dehydrogenase suggest that L-2-HG might play a limited role in the epigenetic reprogramming of early pig embryos. Furthermore, due to limitations in sample size, we were unable to identify specific changes in DNA and histone methylation sites in the *Myc*-overexpressing group, which will be a focus of future studies.

H3K27me3 is considered a key epigenetic mark regulating the entry and exit of ZGA in porcine four-cell embryos. DNA demethylation is crucial for restoring totipotency in fertilized eggs. The commonly observed ZGA delay and incomplete DNA demethylation in SCNT embryos are believed to be major causes of poor development in cloned embryos.

Given that *Myc* is the top differentially expressed transcription factor in both SCNT and IVV embryos, future research will focus on whether transient overexpression of *Myc* at the four-cell stage can enhance the developmental potential of SCNT embryos. This research direction not only holds theoretical significance but also has practical implications for improving cloning techniques.

## 4 Conclusion

The elevated expression of *Myc* in four-cell embryos is crucial for early porcine embryonic development, as it regulates the expression of CS and IDH2 to increase α-KG levels, thereby promoting metabolic activity and demethylation processes.

## 5 Materials and methods

### 5.1 Chemicals

All chemicals used during the experiments were purchased from Sigma-Aldrich (St. Louis, MO, United States), unless otherwise specifically mentioned.

### 5.2 Collection and in-vitro maturation of porcine oocytes

Ovaries were collected from pigs at a local slaughterhouse (Changchun Huazheng, Jilin, China) and transported to the laboratory within 2 h. They were maintained in 0.9% NaCl solution supplemented with 200 IU/mL penicillin and streptomycin at 35°C–36.5°C. Follicular fluid containing cumulus-oocyte complexes (COCs) was aspirated from 3 to 6 mm ovarian follicles using an 18-gauge needle. COCs with at least three layers of cumulus cells were selected and washed three times in manipulation fluid (TCM-199 supplemented with 0.1% polyvinyl alcohol). These COCs were then cultured in *in-vitro* maturation medium (TCM-199 supplemented with 10 μg/mL epidermal growth factor, 0.5 μg/mL porcine LH, 0.5 μg/mL porcine FSH, 26 mM sodium bicarbonate, 3.05 mM glucose, 0.91 mM sodium pyruvate, 0.57 mM cysteine, 0.1% PVA, 10% fetal calf serum, 75 mg/mL penicillin G, and 50 mg/mL streptomycin) for 22–24 h. Subsequently, they were cultured in hormone-free maturation medium (identical to the previous medium but without epidermal growth factor, LH, and FSH) for an additional 20 h at 38.5°C and 5% CO₂ and 95% air. Cumulus cells were removed from oocytes using manipulation fluid supplemented with 0.2% hyaluronidase. Oocytes exhibiting the first polar body (PB1) were considered mature and used for subsequent experiments.

### 5.3 IVF of oocytes and embryo culture

Fresh semen was collected from the Jilin University pig farm and washed by density gradient centrifugation. Briefly, 2 mL of semen was added to Percoll (Solarbio, Beijing, China) in concentrations of 90% and 45%, followed by centrifugation at 300 *g* for 20 min. After removing the supernatant, the sperm pellets were washed with 4 mL Dulbecco’s Phosphate-Buffered Saline (DPBS: 100 mL water with 800 mg NaCl, 20 mg KCl, 112 mg Na₂HPO₄·12H₂O, 20 mg KH₂PO₄, 10 mg CaCl₂, 10 mg MgCl₂·6H₂O, and 100 mg bovine serum albumin (BSA)) and centrifuged at 300 *g* for 10 min. The spermatozoa were resuspended in porcine gamete medium (100 mL water with 0.6313 g NaCl, 0.07456 g KCl, 0.00477 g KH₂PO₄, 0.00987 g MgSO₄·7H₂O, 0.2106 g NaHCO₃, 0.07707 g CaC₆H₁₀O₆·5H₂O, 0.0187 g D-Glucose, 0.3 g PVA, 0.00242 g Cysteine, 0.04504 g C₇H₈N₄O₂, 0.0022 g C₃H₃NaO₃, and 100 μL/mL penicillin and streptomycin). Sixty mature oocytes (1 h post-siRNA injection) were incubated with spermatozoa in 400 μL porcine gamete medium, with a final sperm concentration of 1.6 × 10⁵ to 5.0 × 10⁵ sperm/mL, at 38.5°C, 5% CO₂ and 95% air for 5–6 h. After washing off the adherent sperm, fertilized oocytes were transferred to PZM-3 medium (50 mL water with 0.3156 g NaCl, 0.0373 g KCl, 0.0024 g KH₂PO₄, 0.0024 g MgSO₄·7H₂O, 0.1055 g NaHCO₃, 0.0011 g Na-pyruvate, 0.0308 g Ca-[lactate]₂·5H₂O, 0.0073 g L-glutamine, 0.0273 g hypotaurine, 0.15 g BSA, 1 mL BME amino acid, 0.5 mL MEM non-essential amino acid). Embryos at the two-cell, four-cell, and eight-cell stages were collected after culturing for 24, 48, and 72 h in PZM-3, respectively. The culture medium was changed to PZM-3 with 10% fetal bovine serum after 5 days, and blastocysts were collected after an additional 2 days of culture.

### 5.4 RNA sequencing

The Smart-Seq2 method was used to amplify RNA from each sample (six to eight IVF embryos per group) following the manufacturers instructions. RNA concentration and quality were assessed using a Qubit 2.0 Fluorometer (Life Technologies, CA, United States) and an Agilent Bioanalyzer 2100 system. The amplified cDNA was used for transcriptome library construction. Following the library construction, the insert size was assessed using an Agilent Bioanalyzer 2100 system. The accurate insert size was quantified using a Taq-Man fluorescence probe on an AB Step One Plus Real-Time PCR system (library valid concentration >10 nM). The libraries were then clustered using a cBot cluster generation system and sequenced on an Illumina platform by Zhejiang Annoroad Biotechnology (Beijing, China) to generate 150 bp paired-end reads.

### 5.5 RNA isolation and quantitative PCR

Total RNA was extracted, and complementary DNA (cDNA) was synthesized using the SuperScript™ IV CellsDirect™ cDNA Synthesis Kit (Invitrogen, Carlsbad, CA, United States) following the manufacturer’s instructions. Quantitative PCR was performed using FastStart Essential DNA Green Master (Roche, Basel, Switzerland) on a Step One Plus Real-Time PCR system. Primer sequences are provided in Supplementary Material 1. Data were analyzed using the 2^−ΔΔCT^ method, with GAPDH or β-ACTB as internal reference genes. Each quantitative PCR experiment was repeated three times (See Supplementary Table 1 in the Supplementary Material for primer details).

### 5.6 Immunofluorescence staining

Embryos were washed with PBS containing 0.1% polyvinylpyrrolidone (PVP) and then treated with acidic Tyrode solution (pH 2.5) to dissolve the zona pellucida. After washing, embryos were fixed with 4% paraformaldehyde for 30 min in the dark. Following another wash, embryos were permeabilized with 0.2% Triton X-100/PBS for 20 min and blocked with 2% BSA in PBS for 1 h. For 5 mC/5 hmC staining, embryos were treated with 4M HCl and Tris-HCl for 30 min each, followed by BSA blocking. Primary antibodies were incubated overnight at 4°C, and secondary antibodies were incubated at 37°C for 2 h in the dark. DNA was stained with 10 μg/mL DAPI for 15 min.

Samples were observed under a Nikon Eclipse Ti-U microscope equipped with appropriate filters. Images were captured using a DS-Ri2 CCD camera and analyzed with NIS-Elements BR software. Fluorescence intensity was evaluated using ImageJ software. Background fluorescence intensity was subtracted for further analysis.

### 5.7 Mitochondrial membrane potential, ATP, and ROS assays

The MMP was measured following the protocol provided by Solarbio (CA1310-100, Beijing, China). Four-cell or eight-cell embryos (at least 20 embryos per replicate) were washed three times in PBS-0.1% PVP and fixed with 4% paraformaldehyde for 1 h. After washing, embryos were incubated in PBS-0.1% PVP supplemented with 500 nM BODIPY FL ATP in the dark for 2 h. Fluorescence images were captured using an epifluorescence microscope with excitation and emission wavelengths of 504 nm and 514 nm, respectively.

ROS levels were measured using a ROS assay kit (Beyotime, Shanghai, China). Embryos were incubated in DPBS-0.1% PVA containing 10 μM 2′,7′-dichlorodihydrofluorescein diacetate at 38.5°C in the dark for 15 min. After washing, embryos were placed in microdrops and covered with mineral oil. Fluorescence images were captured using an epifluorescence microscope.

### 5.8 LINE-1 methylation levels in early embryos

LINE-1 methylation levels were detected according to the method described by Xu et al. (2020).

### 5.9 Measurement of α-KG content in early embryos

The α-KG content in early embryos was measured using a Solarbio kit (BC5425, Beijing, China). Briefly, 6,500 four-cell stage embryos were collected, and the zona pellucida was removed using acidic Tyrode’s solution. Embryos were lysed in the kit’s lysis buffer (up to 80 µL) and disrupted by ultrasonic treatment on ice (300 W power, 3-s bursts with 7-s intervals, for a total of 3 min). The lysate was centrifuged at 4°C, and the supernatant was extracted. Reagents were added according to the protocol, mixed thoroughly, and absorbance at 340 nm was measured at 20 s (A1). After incubation at 37°C for 90 min, absorbance at 340 nm was measured again at 90 min and 20 s (A2). Calculations followed the protocol provided with the kit.

### 5.10 Resource identification initiativ

For the detection of *Myc*, we used the *Myc* antibody (Cell Signaling Technology, Cat# 9402S) at a dilution of 1:1,500. The 5 mC antibody (Epigentek, Cat# BI-MECY-0100) was used at a dilution of 1:100. For the detection of H3K27me3, we used the H3K27me3 antibody (Cell Signaling Technology, Cat# C36B11) at a dilution of 1:200. The Alexa Fluor 488 goat anti-mouse secondary antibody (Thermo Fisher Scientific, Cat# A32723) and the Alexa Fluor 488 goat anti-rabbit secondary antibody (Thermo Fisher Scientific, Cat# A-11008) were used at a dilution of 1:200.

### 5.11 Statistical analysis

Data were expressed as mean ± standard deviation. Experiments were repeated at least three times. Statistical analysis was performed using GraphPad Prism 10.1.2 software (GraphPad Software, Boston, MA, United States). Comparisons between two data sets were made using t-tests, while one-way analysis of variance (ANOVA) followed by pairwise comparisons was used for comparisons among multiple data sets. P-value of <0.05 was considered statistically significant.

## Data Availability

The datasets presented in this study can be found in online repositories. The names of the repository/repositories and accession number(s) can be found in the article/Supplementary Material.
