# Supplementary material for: Regulation of α-Ketoglutarate levels by Myc affects metabolism and demethylation in porcine early embryos
Source: Front Cell Dev Biol. 2024 Nov 26;12:1507102. doi: 10.3389/fcell.2024.1507102 (PMC11628527; doi:10.3389/fcell.2024.1507102)
Supplement: Supplementary file 1 [file DataSheet1.docx]

Supplementary Material

# Supplementary Figures and Tables

## Supplementary Figures


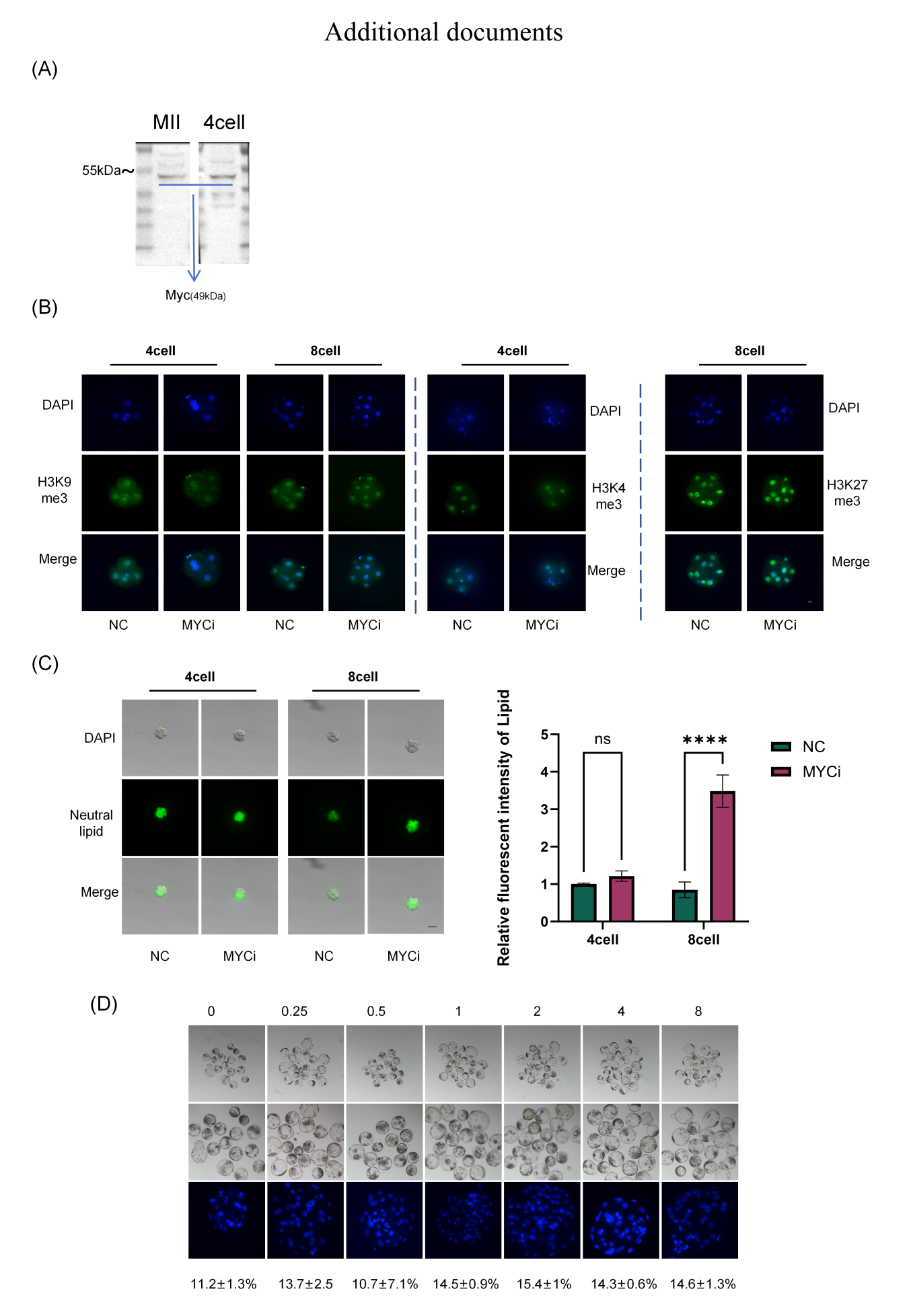


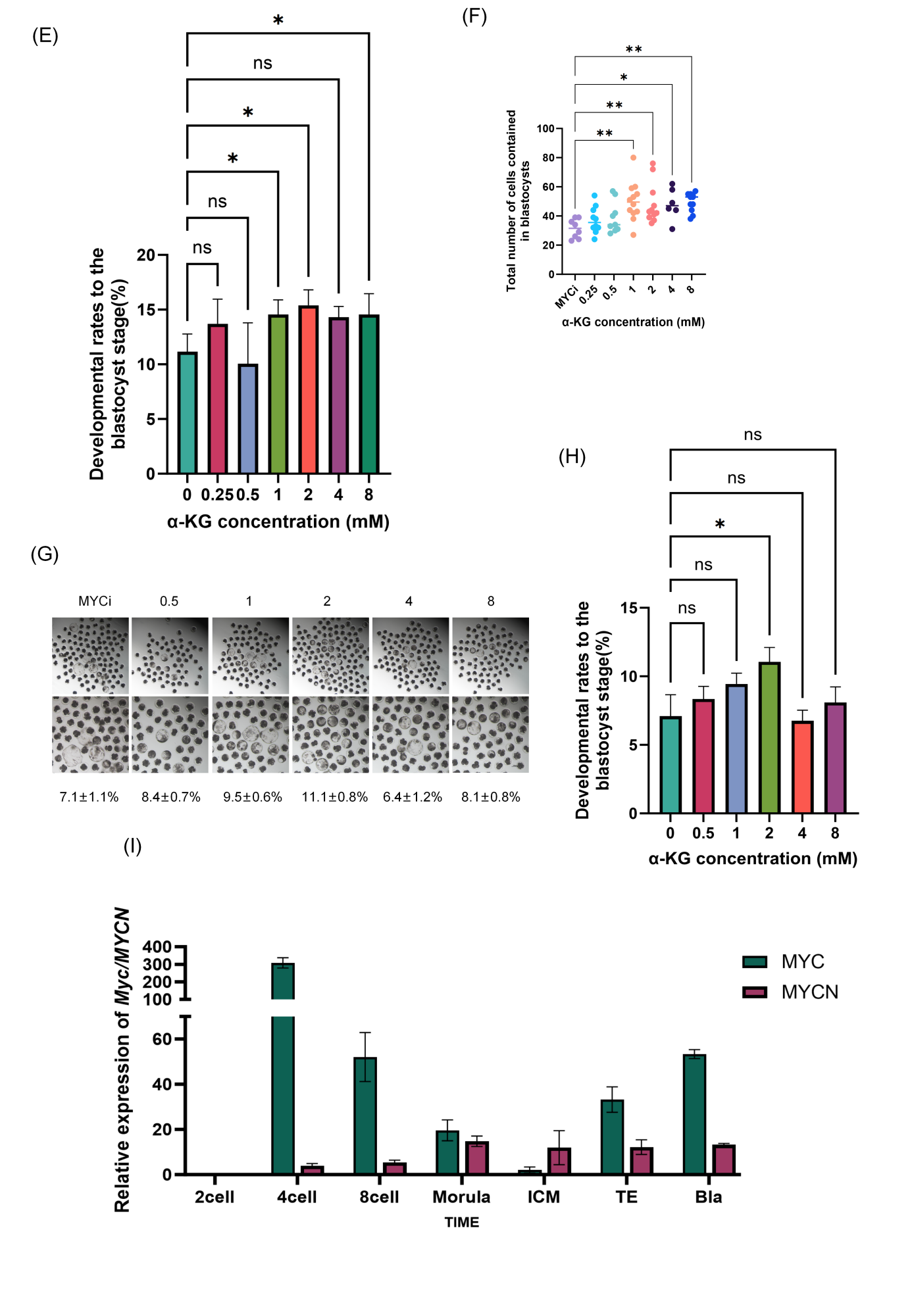


**Supplementary Figure 1.**

1. Western blot analysis of MYC protein, with the predicted molecular weight being 49 kDa.
2. Immunofluorescence staining of H3K4me3,H3K27me3 and H3K9me3 in four-cell or eight-cell embryos treated with DMSO or 10058-F4 for 24 hours (n > 10).

c: Neutral lipid content analysis: At the four-cell stage, the neutral lipid content in the MYCi group was similar to the control group. However, at the eight-cell stage, the MYCi group had significantly higher neutral lipid content compared to the control group. (Scale bar: 100 μm)

1. Embryos were treated with DMSO or 10058-F4 (25 μM) along with various concentrations of α-KG. The blastocyst formation rate and total cell number in blastocysts were measured (n > 100).

   e: Bar graph illustrating the blastocyst formation rate.

f: Total blastocyst cell count in groups supplemented with different concentrations of α-KG compared to the MYCi group treated with 25 μM 10058-F4.

g. Embryos were treated with DMSO or 10058-F4 (50 μM) along with various concentrations of α-KG. The blastocyst formation rate and total cell number in blastocysts were measured (n > 100).

h: Bar graph illustrating the blastocyst formation rate.

i:Expression of myc and MYCN (FPKM)

## Supplementary Table1

| \| **Primer:** \|  \| \| --- \| --- \| \| **IDH2_f** \| GGCTCAGGTCCTCAAGTCTT \| \| **IDH2_r** \| CTCAGCTTCGATGGTCTTGC \| \| **CS_f** \| CTCATGCGCTTGTACCTCAC \| \| **CS_r** \| AAGTAGGGGTCTGAAAGGGC \| \| **LINE1_OF** \| GTTAGGGGGGGGTTTGGTTTTTTAG \| \| **LINE1_OR** \| CTCCCCTTAATCCTATAAAACTTCC \| \| **LINE1-IF** \| TTTGGGAGGTTTTTAAATTATTTGA \| \| **LINE1-IR** \| TATTCTATAAAAACCCACCCCTTCT \| \| ***Myc*-f** \| CAACGTCAGCTTCACCAACA \| \| ***Myc*-r** \| TGGGCAGCAACTCGAATTTC \| \| **GAPDH-f** \| AGGTCGGAGTGAACGGATTTG \| \| **GAPDH-r** \| CCATGTAGTGGAGGTCAATGAAG \| \| **β-ACTB-f** \| CATCGTCCACCGCAAATG \| \| **β-ACTB-r** \| AGCCATGCCAATCTCATCT \| \| **ACO2-f** \| GGAGATGAGAACTACGGCGA \| \| **ACO2-r** \| AGGTTGGTTTCGTGGATCCT \| \|  \|  \| \| **Antibody:** \| item no \| \| ***Myc*** \| 9402S \| \| **5mC** \| BI-MECY-0100 \| \| **H3K27me3** \| C36B11 \| \| **Alexa Fluor 488 goat anti-mouse** \| A32723 \| \| **Alexa Fluor 488 goat anti-rabbit** \| A-11008 \| \|  \|  \| \| **Chemicals:** \|  \| \| **name** \| item no \| \| **10058-F4** \| S7153 \| \| **Dimethyl-α-KG** \| 349631-5G \| \| **BODIPY™ FL ATP** \| A12410 \| \| **H2DCFDA (DCFH-DA)** \| S0033S \| \| **JC-1** \| CA1310 \| |
| --- | --- | --- | --- | --- | --- | --- | --- | --- | --- | --- | --- | --- | --- | --- | --- | --- | --- | --- | --- | --- | --- | --- | --- | --- | --- | --- | --- | --- | --- | --- | --- | --- | --- | --- | --- | --- | --- | --- | --- | --- | --- | --- | --- | --- | --- | --- | --- | --- | --- | --- | --- | --- | --- | --- | --- | --- | --- | --- | --- | --- | --- | --- | --- | --- |

# Data Availability Statement

The datasets generated for this study can be found in the Genome Sequence Archive (GSA) at the National Genomics Data Center (China National Center for Bioinformation / Beijing Institute of Genomics, Chinese Academy of Sciences) under the accession number CRA019376. These data are publicly accessible at the following URL: https://ngdc.cncb.ac.cn/gsa.
